# Metaheuristic Prediction of the Compressive Strength of Environmentally Friendly Concrete Modified with Eggshell Powder Using the Hybrid ANN-SFL Optimization Algorithm

**DOI:** 10.3390/ma14206172

**Published:** 2021-10-18

**Authors:** Seyed Vahid Razavi Tosee, Iman Faridmehr, Chiara Bedon, Łukasz Sadowski, Nasrin Aalimahmoody, Mehdi Nikoo, Tomasz Nowobilski

**Affiliations:** 1 Department of Civil Engineering, Jundi-Shapur University of Technology, Dezful 18674-64616, Iran; vrazavi@jsu.ac.ir; 2 Department of Building Construction and Structural Theory, South Ural State University, Lenin Prospect 76, 454080 Chelyabinsk, Russia; s.k.k-co@live.com; 3 Department of Engineering and Architecture, University of Trieste, 34127 Trieste, Italy; chiara.bedon@dia.units.it; 4 Department of Materials Engineering and Construction Processes, Faculty of Civil Engineering, Wroclaw University of Science and Technology, 50-370 Wrocław, Poland; lukasz.sadowski@pwr.edu.pl; 5 Department of Electrical Engineering, Yazd Branch, Islamic Azad University, Yazd 89168-71967, Iran; mahmoody.elec@gmail.com; 6 Young Researchers and Elite Club, Ahvaz Branch, Islamic Azad University, Ahvaz 68875-61349, Iran; sazeh84@yahoo.com

**Keywords:** eggshell powder concrete, bio-waste material, mechanical properties, artificial neural network, shuffled frog leaping optimization algorithm

## Abstract

The aim of this article is to predict the compressive strength of environmentally friendly concrete modified with eggshell powder. For this purpose, an optimized artificial neural network, combined with a novel metaheuristic shuffled frog leaping optimization algorithm, was employed and compared with a well-known genetic algorithm and multiple linear regression. The presented results confirm that the highest compressive strength (46 MPa on average) can be achieved for mix designs containing 7 to 9% of eggshell powder. This means that the strength increased by 55% when compared to conventional Portland cement-based concrete. The comparative results also show that the proposed artificial neural network, combined with the novel metaheuristic shuffled frog leaping optimization algorithm, offers satisfactory results of compressive strength predictions for concrete modified using eggshell powder concrete. Moreover, it has a higher accuracy than the genetic algorithm and the multiple linear regression. This finding makes the present method useful for construction practice because it enables a concrete mix with a specific compressive strength to be developed based on industrial waste that is locally available.

## 1. Introduction

During the past decade, many countries have focused on various aspects of cleaner production, including the partial reduction of cement when producing concrete [1,2,3]. The partial replacement of cement is nowadays a crucial requirement for sustainable development in the construction industry. Previous literature has presented the use of a wide range of different materials as a partial replacement for cement. Fly ash [4,5], rice husk ash [6,7], palm oil fuel ash [8,9], nano-silica [10,11], and pumice powder [12,13] are some of the replacement materials used in the concrete industry. The utilization of supplementary cementitious materials (e.g., fly ash, rice husk ash, palm oil fuel ash, nano-silica, pumice powder, metakaolin) has had a very positive impact regarding the protection of the environment [14].

Reusing industrial and agricultural waste as an alternative building material significantly improves sustainability in the building sector [15,16]. Nevertheless, the calcium oxide (CaO) content of such material is low, consequently meaning that low-strength concrete products are produced. Among biowastes, eggshells (ES) [17,18], lime [19], sludge furnace [20,21], and oyster shells [22] contain some amounts of calcium in their composition.

Eggshell (ES) is an environmentally friendly biowaste material that can be utilized for cost-effective construction. Since 1999, ES has been widely employed for its calcium for calcium phosphate synthesis applications [17,23]. The food industry is the leading producer of ES. The waste is predominantly transferred to open landfill sites without any treatment, which increases the likelihood of health and environmental hazards because of toxic gas emissions. Eggshell powder can be easily ground into small particles because the process of producing eggshell powder consists of washing, drying, grinding, and sieving.

The physical, mineral, and chemical characteristics of eggshell powder differ from those of cement. Hemalatha et al. [24] combined ES with high-volume fly ash (HVFA) to improve the low strength of FA concrete. The calcium carbonate (CaCO_3_) in the ES was extracted, which in turn accelerated the hydration in the FA concrete. Incorporating CaCO_3_ in HVFA concrete causes carbo-aluminates to be formed, which result from the interaction of the carbonate ion from the Calcium-chloro (CC)with the aluminate hydrate from the Portland cement hydration [25,26]. The existing CaCO_3_ enhances compressive strength because of the stabilization of ettringite and mono-carbonate [27,28].

Concrete modified with eggshell has low radioactive permeability. Binici et al. [29] investigated eggshell powder as a protection layer for buildings against the effects of external radiation. Such concrete products can be utilized as radiation insulators in walls. The results revealed that eggshell powder in various proportions reduces the compressive and flexural strengths of mortar at all curing ages.

Eggshell powder and cement are similar in their CaCO3 content. Ashok et al. [30] reported that typical eggshells contain 90.5% calcium carbonate, 6.8% calcium hydroxide, and 0.7% calcium oxide. The large share of calcium carbonate in eggshells makes them a biocompatible material. Eggshells have impressive physical and mechanical properties (i.e., compressive strength, tensile strength, and low water absorption) [6]. Pliya and Cree [31] studied the properties of white and brown chicken eggs as a replacement for conventional limestone in cement mortars. They established that adding limestone materials to concrete affected its compressive and flexural strengths due to the formation of additional S–C–H gels. Oluwatuyi et al. [32] scrutinized ES as a potential stabilizing material for subgrade soil in highway constructions. Mabah et al. [33] found that recycling eggshell powder and rice husk ash (RHA) to produce an additive for concrete products substantially improves the microstructural and mechanical properties of geopolymers. Jhatial et al. [34] enhanced the pozzolanic reaction with POFA and cement by utilizing eggshell powder in order to increase CaO levels. Amu et al. [35] studied the suitability of eggshell powder as a stabilizer for improving the properties of soil. Hassan et al. [18] studied uncarbonized and carbonized eggshell particulates in polyester composite, and computed their tensile, flexural, compressive, density, impact, and hardness properties. K. Nandhini and J. Karthikeyan [36] investigated self-compacting high-performance concrete containing eggshell powder as a partial cement replacement. They replaced ordinary Portland cement (OPC) with 10%, 20%, and 30% of eggshell powder. The workability tests showed that 10% of eggshell powder-blended-SCC offered higher flowability, and also that the flowing time through a V-funnel consumed a lot of time for large contents of eggshell powder. A higher eggshell powder content resulted in a higher blocking ratio. An examination of the resulting concrete showed that a higher eggshell powder content reduced both the compressive and flexural strength properties.

The compressive strength of concrete is the most common performance measure used by engineers when designing buildings and structures. Compressive strength tests are generally carried out 7–28 days after concrete casting, which in turn leads to delays in the construction process. Failure to perform concrete strength tests may later cause complications, especially in the case of large and complex structures. Therefore, a reliable and fast prediction of compressive strength is essential for quality control, even in the design phase. In addition, the early prediction of compressive strength is important for project scheduling, estimating the time needed to open the concrete formwork, and quality control.

Due to the fact that the relationship between compressive strength and the components of a mix is non-linear (mainly because of its heterogeneous characteristics), mathematical modeling methods have become very complicated. Moreover, the existing empirical equation in international standards estimates compressive strength based on experimental tests. In turn, there has been a lot of research conducted that concerns estimating the compressive strength of concrete produced with industrial byproducts. Since adjusting the composition of a mix is important for optimizing the mechanical properties, cost, and sustainability of concrete made by industrial byproducts, a significant amount of research has been carried out using experimental tests, in turn causing a loss of resources, materials and time. Therefore, a novel modeling system that is not dependent on experimental tests, but which is able to estimate compressive strength with a high accuracy, should be developed.

In recent years, artificial intelligence (AI) systems have been increasingly used for solving regression and classification problems. This is due to the fact that more reliable results are obtained than when using conventional methods [37,38,39,40,41,42,43,44,45]. AI systems have generally shown a lot of potential for solving real-life tasks, particularly non-linear problems. Material science has been a field of significant growth through the application and testing of novel computational models that are able to estimate the mechanical properties of concrete mixtures. Considering the concrete industry, where a minimum of three specimens should be tested at each selected age to measure compressive strength, AI based models can help to avoid the use of such destructive tests. AI-based models allow for the processing of raw experimental data and the identification of the functional relationships between them, even when these relationships are unknown and difficult to identify. These models may be used to estimate the compressive strength of concrete that contains by-products/waste. In the case of large and diverse databases, AI-based models can be used as a reliable tool to validate and verify the existing empirical models that are used in international standards. Table 1 illustrates the most commonly used learning algorithms applied by researchers to estimate the compressive strength of various types of mix designs.

As is indicated in the literature, far too little research has been done on the application of ES in concrete as a source of cement replacement. At the first stage of the current research, the physical and chemical properties of ES, and the properties of the resulting concrete products made from the biowaste, are examined. In addition, to find the optimum percentage of ES in concrete, 16 different mix designs containing various percentages of ES (substituting cement by 0.01% to a maximum of 15%) were prepared, with their compressive strength being tested at different ages. Over the years, several experimental studies have been carried out to examine the effect of eggshell powder on the mechanical properties of concrete. However, there is still a lack of systematic research about using AI approaches to predict the compressive strength of eggshell powder mix designs. The reliable prediction of compressive strength can save time and reduce cost by rapidly generating the required design data. Therefore, in the second phase of the research, using the experimental test database thus generated, an optimized artificial neural network (ANN) combined with the shuffled frog-leaping algorithm (SFL-ANN) was developed to estimate the compressive strength of mix designs. In recent years, SFL-ANN have been applied toward different engineering problems, including soil shear strength simulation [67], predicting river streamflow time series [68], and optimal design of truss structures [69]. This study therefore contributes to current research concerning estimating compressive strength of concrete, because it presents the use of a novel biowaste material for sustainable development in the construction industry. The resulting product offers several benefits, including environmental-friendliness, the reduction of cement usage, and thus, a decrease in energy consumption and greenhouse emissions.

## 2. Properties of Eggshell Powder

Eggshell is an inorganic material containing three different layers—mammillary, calcareous, and cuticle. Eggshells largely encompass CaCO3 (almost 94% of its total weight), organic matter (about 4%), calcium phosphate (around 1%), and magnesium carbonate (about 1%) [70]. Using XRF analysis, Mabah et al. [71] studied the chemical composition of eggshell powder and found that CaO (93.2%) holds the highest percentage among the constituents. According to Okonkwo [72], ESs constitute about 93.70% CaCO3, 4.20% organic matter, 1.30% magnesium carbonate, and 0.8% calcium phosphate by weight. Bashir et al. [73] reported the chemical composition of eggshell powder as 94%, 1%, and 1% of CaCO_3_, CaPO_4_, and MgCO_3_, respectively. Moreover, according to Babu and Neeraja [74], a boiled ES mixture comprises roughly 79% and 19% of CaO and SiO_2_, respectively. Table 2 summarizes the chemical analysis of eggshell powder using the XRF method.

Table 3 summarizes the chemical properties of the Portland cement type I used in this study (Khuzestan Cement Company, Khuzestan Province, Iran). The ordinary Portland cement used in this study (Type I) has a surface area between 3000 and 3500 cm^2^/g. The ASTM specifies a minimum surface area of 2800 cm^2^/g (as determined by the air permeability test, i.e., Blaine) for all types of Portland cement. Jhatial et al. [23] concluded that the fineness of eggshell powder has a significant influence on the compressive strength of concrete. They stated that 50-micron eggshell powder performed better in compressive strength development over the curing period.

The physical properties of ES vary according to the origin of the egg from which it comes. Eggshell powder specific gravity is less than cement, and ranges between 3.15 and 3.18 [75]. The physical properties of eggshell powder vary significantly, depending on its source, preparation, and fineness grade. Earlier studies examined the properties of eggshell powder and determined a 0.85 to 2.66 range for specific gravity [76]. In turn, the bulk density of eggshell powder varies between 700 to 2088 kg/m^3^, while that of cement ranges between 1000 and 1300 kg/m^3^ [77].

## 3. Mixing Proportions of Concrete Containing Eggshell Powder

**Figure 1:**
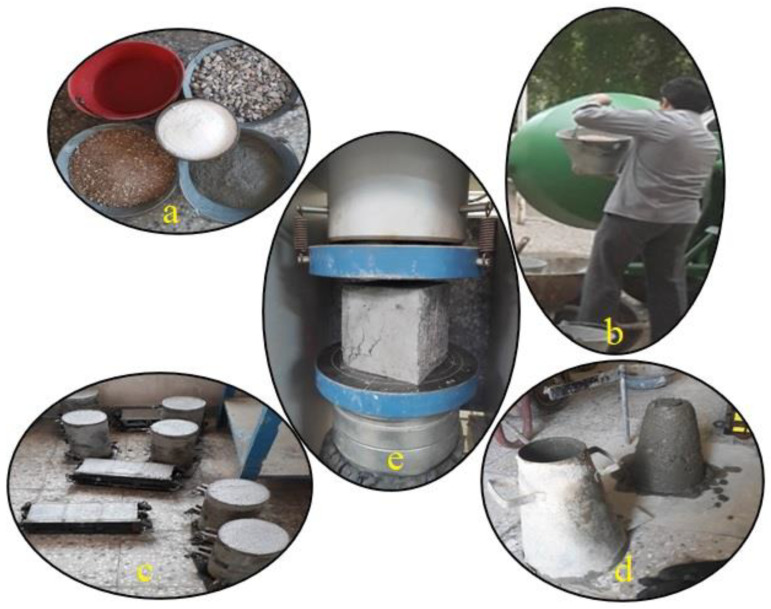
The casting and testing of the mechanical properties concrete modified with eggshell powder: (**a**) mix design, (**b**) mixing the concrete, (**c**) casting the concrete in a mold, (**d**) slump test, and (**e**) compressive strength test.

To examine the compressive strength of concrete samples made using different percentages of eggshell powder as a substitute for cement, cubic samples with dimensions of “10 cm × 10 cm × 10 cm” were used. The evaluation of the mechanical properties of the specimens, the analysis of the fresh concrete’s slump, and the analysis of the compressive strength of the concrete after 3, 7, 14, 28, 90 and 180 days were performed according to ASTM C 143 [78] and ASTM C 39 [79] guidelines, respectively. To prepare the concrete mixes, the calculated amounts of cement, eggshell powder, and fine and coarse aggregates were weighted and mixed for more than 60 s. Water was then added, and the mixing was continued for 180 s. Figure 1 shows the mixing, casting, slump test, and compressive test process of this particular mix design.

The concrete mix, prepared with 100% OPC and natural aggregates, was adopted as the control sample in order to evaluate the performance of the eggshell powder modified concrete. In the eggshell powder modified mix designs, the eggshell powder was used to replace the cement by 1% to a maximum of 15%, with an incremental ratio of 1%, as shown in Table 4. The water to cement ratio was fixed to 0.45 for all the mix designs.

## 4. Test Results

**Figure 2:**
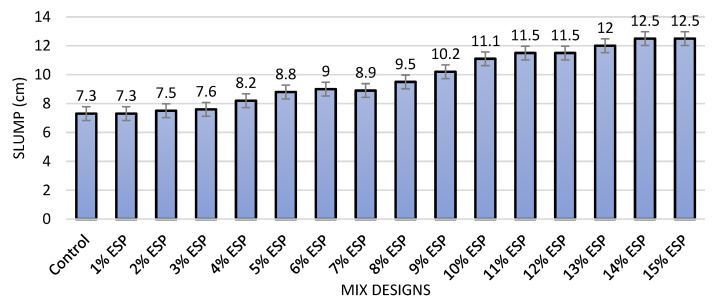
Slump results for all the mix designs.

Figure 2 shows the slump test results for all the studied mix designs. The results indicate that although the slump was remained constant when substituting cement with 1 to 3% of eggshell powder, the slump was increased for the replacement of eggshell powder beyond 4%. The highest decrease was recorded for the specimens containing 14 and 15% of eggshell powder (12.5 cm), while the average slump was recorded for the specimens containing 4 to 15% of eggshell powder is 10.5 cm. Such a slump is suitable for many applications in the concrete construction industry, including columns and walls.

**Figure 3:**
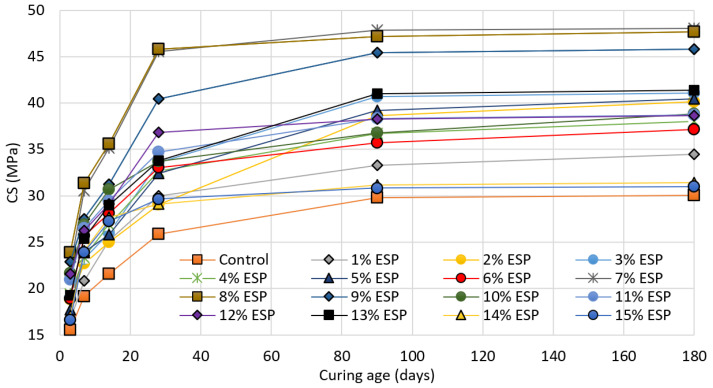
Compressive strength development in the studied mix designs.

Figure 3 depicts the compressive strength values of all the mix designs investigated in this study. The specimens were tested after curing for 3, 7, 14, 28, 90, and 180 days. The results indicated that the highest compressive strength was achieved by the mix containing 7 to 9% of eggshell powder (an average of 46 MPa). This means that the obtained strength was 55% higher than that of the reference specimen. The lowest compressive strength among the eggshell powder modified concretes was achieved by the mix designs containing 1 and 15% of eggshell powder (34.4 and 30.9 MPa, respectively). Moreover, in the case of the specimens containing 10 to 15% of eggshell powder, the average compressive strength was recorded to be equal to 38 MPa. Overall, the results confirm that in all cases the eggshell powder modified concrete provided higher compressive strength when compared to the control specimen.

## 5. Developing the Model to Estimate Compressive Strength

In order to develop a reliable model for estimating compressive strength, it is necessary to explain the physical phenomena, key mechanical parameters and mechanisms that occur in the binder. Conventional approaches for modeling and optimizing complex structure systems and problems require enormous amounts of computing resources, while ANN-based solutions can regularly provide alternatives for efficiently solving problems. Concrete is a non-linear material; therefore, there exists a complicated relationship between the various factors influencing the properties of the mix. Therefore, to predict compressive strength, conventional approaches such as regression analysis are unreliable. Such issue motivates many studies concerning artificial intelligence (AI) in evolutionary or hybrid systems to develop a reliable and effective model that can predict concrete compressive strength.

This study investigates the development of an information model based on a hybrid ANN coupled with the metaheuristic shuffled frog leaping optimization algorithm (SFL-ANN). An ANN is a data processing system that learns from experience and which can generalize its knowledge to new data that is unfamiliar to the model [80,81]. Inspired by the structure of the biological brain, an ANN comprises a group of neurons that operate locally to solve a particular problem. Neural networks acquire knowledge through learning via a simplified human brain-like approach in customary computations that capture the underlying mechanisms in a dataset. The multilayer feed-forward network used in this study is a reliable and commonly used ANN architecture. The multilayer feed-forward network comprises three types of layers, including the input layer (the model’s point of data entry), the hidden layer(s) (in which data processing takes place), and the output layer (though which the network delivers the output results). Every layer comprises a set of nodes denoted as neurons. These neurons are connected to other neurons from the preceding and succeeding layers. The output and the hidden-layer neurons are comprised of three parts: activation function, weights, and biases. Nonlinear sigmoid functions (logsig, tansig) and linear functions (poslin, purelin) are among the most frequently used activation functions [82]. Training algorithms aim to optimize the weight and bias values by minimizing the error function. The backpropagation (BP) algorithm is one of the most reliable and widely used ANN training algorithms [83,84].

### 5.1. Short Description of the Shuffled Frog-Leaping Algorithm (SFLA)

The SFLA algorithm is founded on the memetic evolution, which is inspired by an army of frogs searching for food. It combines the qualities of the social intelligence-based PSO algorithm and the genetic evolution-based memetic algorithm. In the SFLA, a population of frogs denotes a set of probable solutions. The frog group is broken down into several memeplexes, each representing a diverse culture. Knowing that frogs are inclined to encircle the finest frog, which might be a local optimum, some of the members of a memeplex are classified as a sub-memeplex in order to circumvent their convergence to the local optimum. The frog with the weakest position must evolve. The memeplexes are shuffled as a population after a specific number of memetic iterations. The local search and shuffling procedure continue until the solution satisfies the needed index, or until the evolution generations are completed.

**Figure 4:**
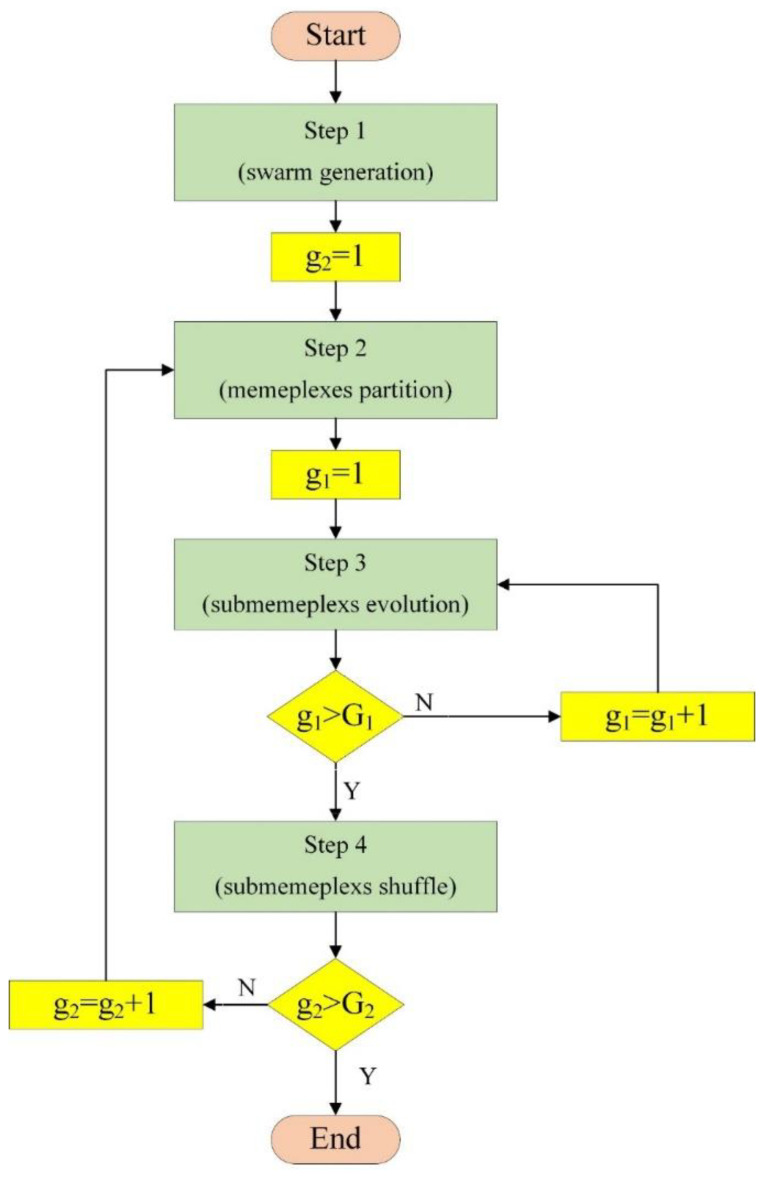
Flowchart of the shuffled frog-leaping algorithm.

The implementation flow of the SFLA is shown in Figure 4.

### 5.2. The Preparation of Training and Testing Data Sets

A dataset consisting of 15 mix designs was developed, as discussed in Section 3, to investigate the compressive strength of the modified concrete containing different percentages of eggshell powder at different ages (varying from 1 to 180 days). The independent input parameters are the following: eggshell powder content; cement content; curing age; and slump, which form a 4 × 1 matrix, while the dependent output parameter (compressive strength (fc’)) is a scalar value (1 × 1 matrix). The minimum and maximum values for each input variable are presented in Table 5.

**Figure 5:**
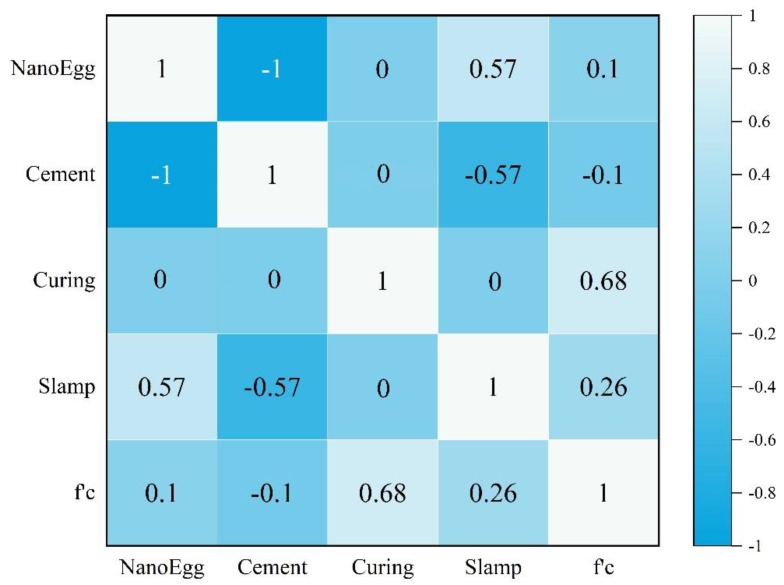
Correlation matrix for the input and output variables.

In statistics, any statistical relationship, whether causal or not, between two random variables is called a correlation or dependency. In the broadest sense, a correlation refers to the degree to which a pair of parameters are linearly associated. A correlation matrix is a table that provides the correlation coefficients among the various input variables. Figure 5 shows the correlation matrix developed for the input variables in this study. A correlation matrix is a table showing correlation coefficients between variables. Each cell in the table shows the correlation between two variables. A correlation matrix is used to summarize data, as an input into a more advanced analysis, and as a diagnostic for advanced analyses. The line of 1.00 s going from the top left to the bottom right is the main diagonal, which shows that each variable always perfectly correlates with itself. Considering the range of data for each variable, and in order to avoid any divergence in the results, the variables were initially normalized within the −1 to 1 range using:

(1) $$X_{n}=\frac{2\left(X-X_{min}\right)}{X_{max}-X_{min}}-1$$

where *X* is the non-normalized value of the variable; *X_n_* is its normalized value in the [−1, 1] range, and *X_max_* and *X_min_* are its maximum and minimum values, respectively.

Figure 5 indicated that (i). cement and ESP are negatively correlated with a value of −1; (ii). ESP and cement have the greatest influence on slump compared to other parameters; (iii). The curing parameter has the most effect on the compressive strength of concrete and is in line with it in terms of vector, but the cement parameter has the least effect and in terms of vector in the opposite direction.

**Figure 6:**
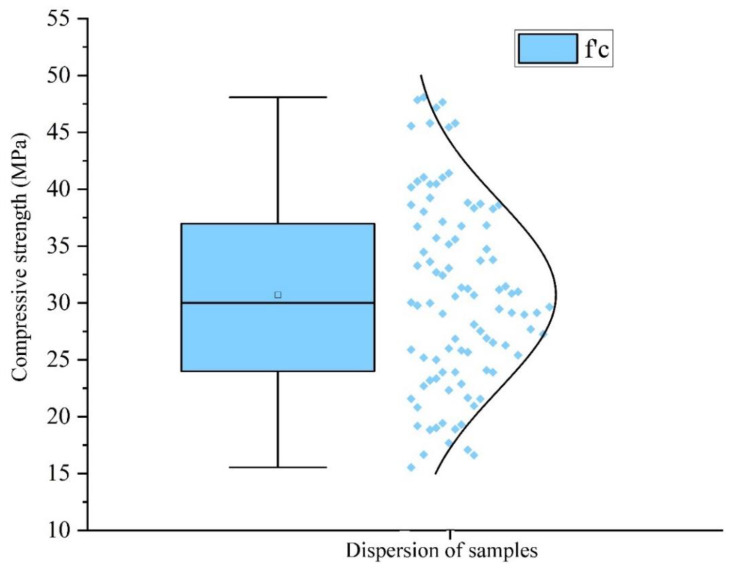
Distribution plot for the input parameter (compressive strength).

Since the statistical behavior of the output parameter (compressive strength) should be evaluated, a distribution plot was constructed, as shown in Figure 6. The “bell curve” shown in Figure 6 originates from the fact that the graph used to depict a normal distribution consists of a symmetrical bell-shaped curve. This plot reveals that the compressive strength was mainly distributed in the range of 25 MPa to 35 MPa.

A trial-and-error method is often used to obtain the most efficient ANN model architecture that best reflects the characteristics of the experimental test data. In the present study, the hidden layers’ neurons are determined according to Equation (2) [85]:

(2) $$N_{H}\leq \min\left(2N_{I}+1;\frac{N_{TR}}{N_{I}+1}\right)$$

where *N_H_* denotes the number of neurons in the hidden layers, *N_I_* is the number of input variables, and *N_TR_* is the number of training samples in the database.

Considering that we have four input variables, the mentioned Equation computed less than nine neurons for the hidden layers. Therefore, several networks with different topologies, with a maximum of two hidden layers and 9 neurons at each hidden layer, were trained and examined in this study. We utilized the hyperbolic tangent transfer function and the Levenberg–Marquardt training algorithm for all the networks. Moreover, the statistical indices, including the average absolute error (*AAE*), coefficient of determination (*R^2^*), variance account factor (*VAF*), and *MSE*, which are expressed in Equations (3)–(6), were employed to assess the efficiency of the various topologies:

(3) $$AAE=\frac{\left|\sum_{i=1}^{n}\frac{\left(O_{i}-P_{i}\right)}{O_{i}}\right|}{n}$$

(4) $$R^{2}=\frac{\sum_{i=1}^{n}(y_{i\left(model\right)-}{\bar{y}}_{\left(Actual\right)})}{\sum_{i=1}^{n}(y_{\left(Actual\right)}-{\bar{y}}_{\left(Actual\right)})}$$

(5) $$VAF=\left[1-\frac{var\left(O_{i}-P_{i}\right)}{var\left(O_{i}\right)}\right]$$

(6) $$RMSE={\left[\frac{1}{n}\sum_{i=1}^{n}{\left(P_{i}-O_{i}\right)}^{2}\right]}^{\frac{1}{2}}$$

In total, 12 different network topologies were examined. It was found that the network with a topology that consists of 4–4–3–1-layer architecture obtained the lowest values of errors for *MSE* and *AAE*, and the highest values of *VAF*. This network is the best for estimating the output parameters (compressive strength)—as shown in Table 6. The ANN used in this study was the Newff Feed Forward, where 70% of the experimental data was assigned to training, with the remaining 30% being used for network testing.

**Figure 7:**
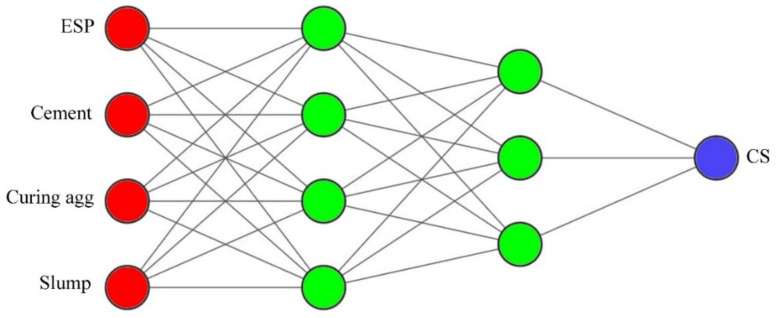
Proposed 4–4–3–1 topology of a feed-forward neural network.

Figure 7 illustrates the proposed 4–4–3–1 topology of the feed-forward neural network with two hidden layers, four input variables (neurons), and one output parameter.

The SFLA optimization algorithm was used to provide the least prediction error for the trained structure and to optimize the ANN’s weights and biases. The properties of the SFLA algorithm’s parameters are given in Table 7 [86].

### 5.3. Multiple Linear Regression and Genetic Algorithm Models

To validate the proposed hybrid SFLA-ANN model used in this study, a multiple linear regression (MLR) model and a genetic algorithm combined with an ANN (GA-ANN) were also developed. In an MLR model, two or more independent variables significantly affect the dependent variable, as shown in the following Equation:

(7) $$y=f\left(x_{1},x_{2},\ldots \right)\to y=a_{0}+a_{1}x_{1}+a_{2}x_{2}+\ldots$$

where *y* is a dependent variable; *x*_1_, *x*_2_, …, are independent variables, and *a*_1_, *a*_2_, … are coefficients of the Equation.

The following Equation shows the most suitable coefficients for the MLR model for estimating the overall deflection of the studied specimens.

(8) compressive strength=200.5−0.293×ESP+0.001×Cement+0.8839×Curing age+8.75×Slamp

A genetic algorithm combined with an ANN (GA-ANN) was implemented for the second evaluation. Its characteristics are summarized in Table 8.

### 5.4. Comparison of the Accuracy of Informational Models

**Figure 8:**
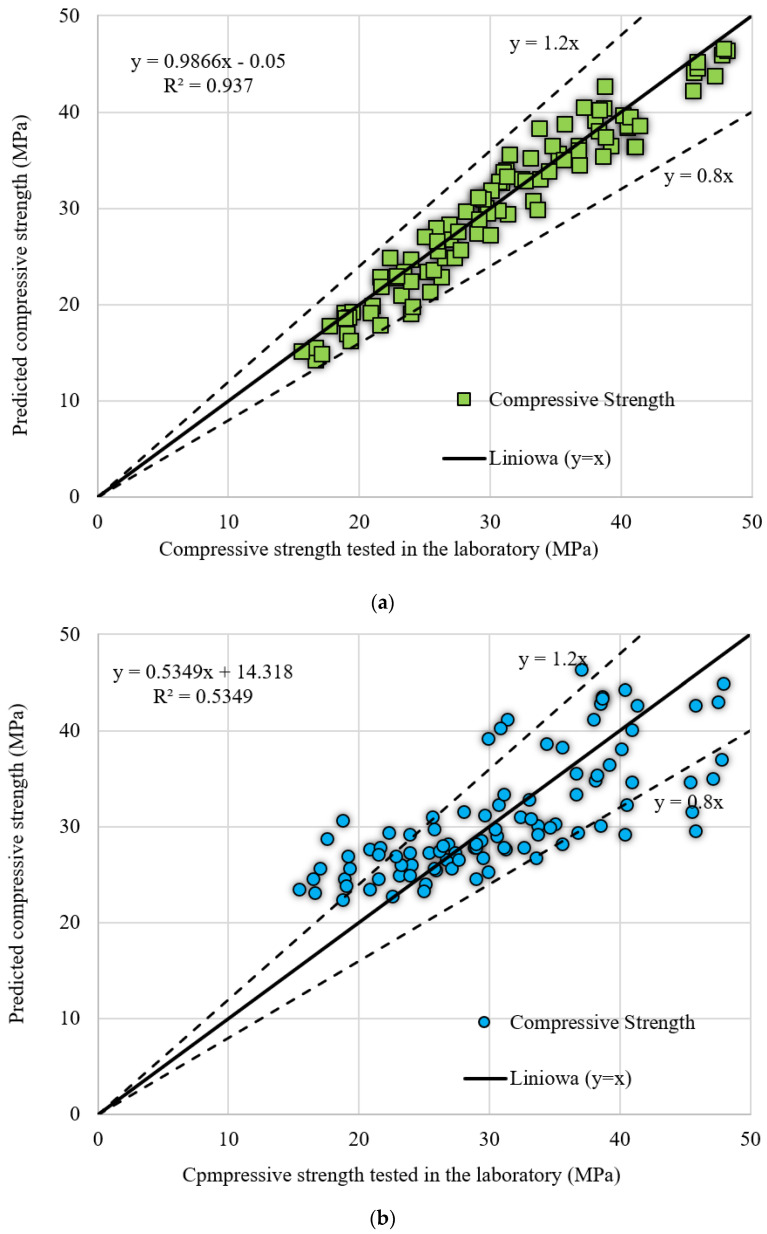

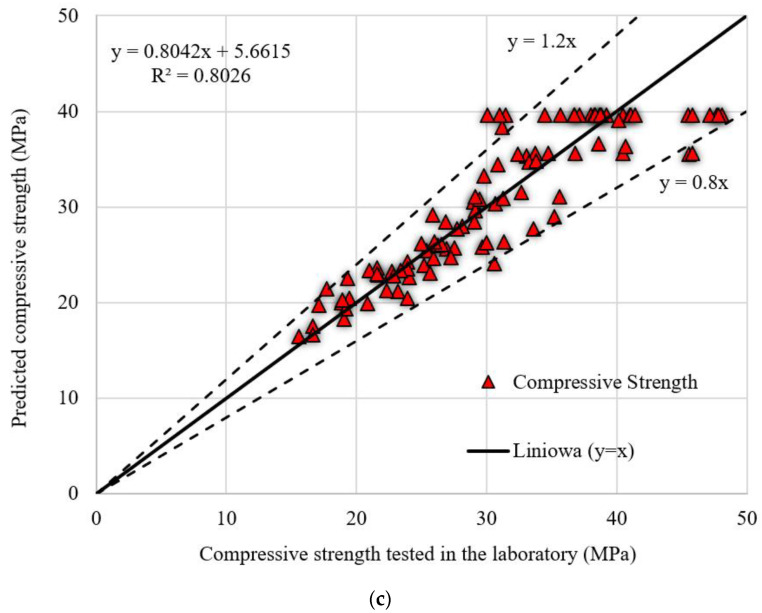
Comparison between the experimental and informational models for the compressive strength of eggshell powder modified concrete (**a**) SFLA-ANN, (**b**) MLR, and (**c**) GA-ANN.

Figure 8 shows the comparison between the actual experimental data and the results obtained on the basis of the developed models: SFLA-ANN, MLR and GA-ANN. The figure also indicates that the hybrid SFLA-ANN model provided more reliable estimations of the overall deflection of RC beams when compared to that of the international standards and the MLR and GA-ANN models.

Table 9 shows the statistical metrics for all the informational models in the case of the training and testing of all the data. The results indicate that the proposed hybrid SFLA-ANN model yielded the most reliable results for the compressive strength estimation of eggshell powder modified concrete. The maximum values of the *VAF* and average of the hybrid SFLA-ANN model were closest to unity, indicating its robust predictive capability.

**Figure 9:**
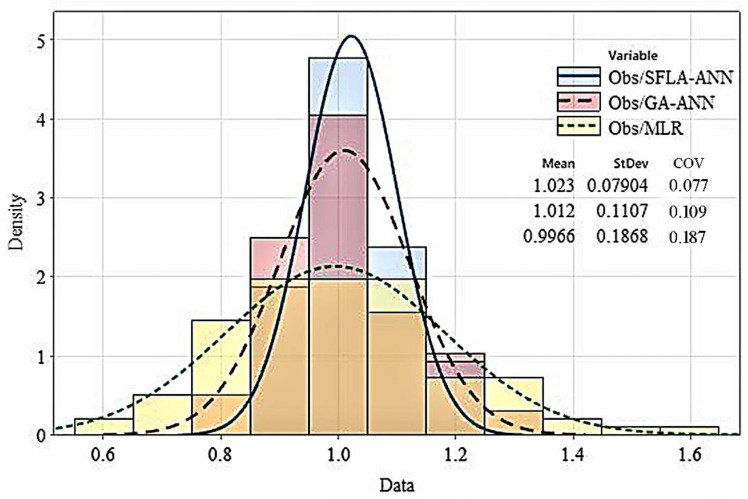
Histogram of the test-to-prediction ratio.

When analyzing the histogram, it can be seen that the distribution of the SFLA-ANN hybrid model is less dispersed than in the case of the GA-ANN and MLR models. In addition, the COV and STDEV values, which were determined for the SFLA-ANN model, are equal to 0.077 and 0.079, respectively (Figure 9). The low StDev estimated by the proposed SFLA-ANN model indicates that the $CS_{exp}$/$CS_{\;theoretical}$ values tend to be close to the mean and less spread out over a wider range.

**Figure 10:**
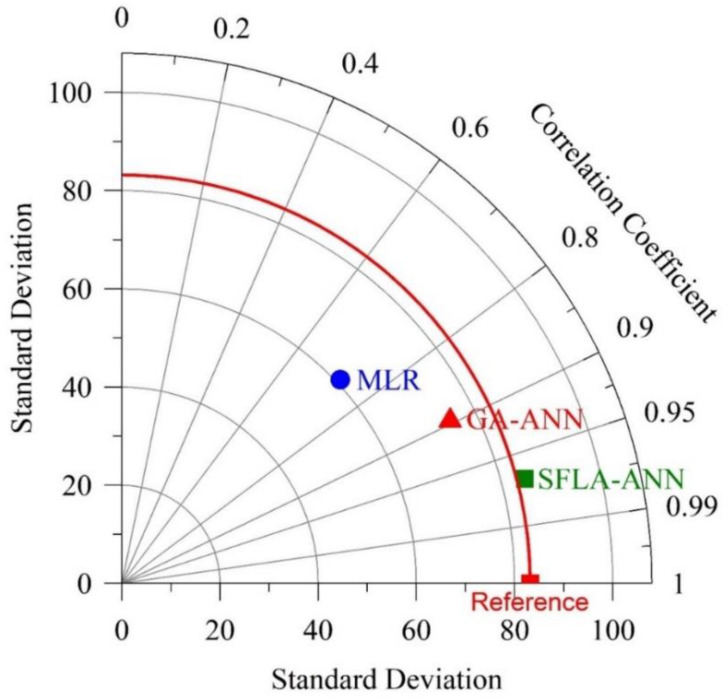
Taylor Diagram visualization of the SFLA-ANN model’s performance in predicting compressive strength.

Figure 10 shows another visual representation (Taylor diagram) of the comparison of the performance of the hybrid SFLA-ANN model against the other information models. This diagram depicts a graphical illustration of the adequacy, based on the centered root-mean-square (RMS) difference, StDev, and correlation coefficient, of each of the investigated models. The results indicate that the best prediction of compressive strength can be obtained in the case of the SFLA-ANN model. The MLR model resulted in higher values of the root mean-square-centered difference and StDev, indicating the rather low accuracy of the model in estimating the experimental data when compared to the GA-ANN model.

## 6. Concluding Remarks

This study examined the mechanical properties of concrete modified with eggshell powder. By following existing literature and by performing an electron microscopy test, the chemical and physical properties of eggshell powder were examined. Subsequently, in order to find the optimum percentage of eggshell powder in concrete, 16 different mix designs containing various percentages of eggshell powder were prepared, and their compressive strengths were tested at different ages. The eggshell powder substituted the cement by 0.01% to a maximum of 15%. Finally, using the experimental test database thus generated, an optimized artificial neural network (ANN) combined with the shuffled frog-leaping algorithm (SFLA-ANN) was developed to estimate the compressive strength of the mix designs. The most important findings of the conducted research include:

1. The highest slump (12.5 cm) was recorded for the mix design containing 14 and 15% of eggshell powder, while the average slump for the specimens containing 4 to 15% of eggshell powder was 10.5 cm.
2. The highest compressive strength was achieved for the mix containing 7 to 9% of eggshell powder (an average of 46 MPa at the age of 28 days); this result is 55% higher than that of the control specimen. However, the lowest compressive strength among the eggshell powder modified concretes was achieved by the mix designs containing 1 and 15% of eggshell powder-34.4 and 30.9 MPa, respectively. Such results are in compliance with those of Yerramala [75], who concluded that a 5% ESP replacement would be optimal for obtaining the maximum compressive strength in concrete.
3. Various statistical metrics were deployed to compare the actual compressive strength of eggshell powder modified concrete with the corresponding values predicted by the diverse information models. The results confirmed that the proposed SFLA-ANN model attained the most reliable and robust results for determining the compressive strength of eggshell powder modified concrete.
4. The obtained histogram of the test-to-prediction ratios indicated that the SFL-ANN model is better fitted and less dispersed than the other models. Such a result indicates that the proposed information model can achieve a more accurate, safer, and more reliable estimation of the compressive strength of eggshell powder modified concrete.
5. It is recommended to compare the life cycle of eggshell powder modified concrete against OPC-based concrete. The boundary of the cradle-to-gate system should be extended to include the mechanical and durability properties of mix designs. By using this approach, not only is the impact of material manufacturing accounted for, but the impacts of service life are also incorporated in the life cycle assessment criteria.

## Figures and Tables

**Table 1:** Commonly used learning algorithms applied by several researchers to estimate compressive strength.

Type of Learning Algorithm	Admixture and Concrete Feature	
	Below 60 MPa	Over 60 MPa
Levenberg-Marquardt	Kostic and Vasovic (2015) [46]; Basic Concrete Asteris et al. (2021) [47]; Cement Mortar Tanyildizi (2018) [48]; Carbon Fiber-Reinforced Lightweight Concrete Liang et al. (2018) [49]; Basic Concrete	Ghafari et al. (2015) [50]; Ultra High Performance Concrete Chitra et al. (2016) [51]; High Performance Concrete
Broyden-Fletcher-Goldfarb-Shanno	Kao et al. (2018) [52]; Pozzolanic Concrete	–
Genetic Algorithm	Nikoo et al. (2015) [53]; Basic Concrete Yuan et al. (2014) [54]; Basic Concrete Heidari et al. (2017) [55]; Green Concrete	Cheng et al. (2014) [56]; High Performance Concrete
Gene Expression Programming	Kiani et al. (2016) [57]; Preformed-Foam Cellular Concrete Hadianfard and Jafari (2016) [58]; Lightweight Aggregate Concrete	–
Bayesian	Paul et al. (2018) [59]; Recycled Aggregate	–
Grey Wolves	Mahdi Shariati et al. (2020) [60]; Green Concrete	Behnood et al. (2018) [61]; Silica Fume Concrete
Firefly Algorithm	–	Bui et al. (2018) [62]; High Performance Concrete
Particle Swarm Optimization	Tsai et al. (2016) [63]; Basic Concrete	–
Imperialist Competitive Algorithm	Sadowski et al. (2018) [64]; Basic Concrete	–
Model Tree Algorithm	–	Behnood et al. (2017) [65]; High Performance Concrete
Whale Optimization Algorithm	Dieu Tien Bui et al. (2019) [66]; Basic Concrete	

**Table 2:** The chemical analysis of eggshell powder using the XRF method.

Compositions	Weight Percentage	Compositions	Weight Percentage
Na_2_O	0.32	MgO	0.74
S	0.2	K_2_O	0.06
SrO	0.2	SiO_2_	18.09
CaO	54.89	P_2_O_5_	0.31
Fe_2_O_3_	0.04		

**Table 3:** Average chemical composition of Portland cement type I.

Compositions (wt%)			
CaO	SiO_2_	Al_2_O_3_	Fe_2_O_3_
63.8	22.1	5.0	3.0

**Table 4:** Concrete mix design.

Mix Design	Cement (kg/m^3^)	Eggshell Powder (kg/m^3^)	Fine Aggregate (kg/m^3^)	Coarse Aggregate (kg/m^3^)
Control	450	0	563	672
1	445.5	4.5	563	672
2	441	9	563	672
3	436.5	13.5	563	672
4	432	18	563	672
5	427.5	22.5	563	672
6	423	27	563	672
7	418.5	31.5	563	672
8	414	36	563	672
9	409.5	40.5	563	672
10	405	45	563	672
11	400.5	49.5	563	672
12	396	54	563	672
13	391.5	58.5	563	672
14	387	63	563	672
15	382.5	67.5	563	672

**Table 5:** Input and output variable properties.

Statistical Index	Unit	Type	Max	Min	Average	STD
Eggshell Powder (ESP) Content	kg/m^3^	Input	67.5	0.0	33.8	20.9
Cement Content	kg/m^3^	Input	450.0	382.5	416.3	20.9
Curing Age	Day	Input	180.0	3.0	53.7	63.9
Slump	cm	Input	12.5	7.3	7.9	2.9
fc’	MPa	Output	48	15.5	30.7	8.3

**Table 6:** Statistics related to 12 ANNs (the SFLA algorithm-training and testing data).

Num.	Hidden Layer 1	Hidden Layer 2	Train			Test		
			*MSE*	*AAE*	*VAF*	*MSE*	*AAE*	*VAF*
1	3	3	0.005	0.002	92	0.013	0.003	86
2	3	4	0.028	0.033	53	0.042	0.029	53
3	3	5	0.027	0.044	55	0.042	0.037	52
4	4	3	0.006	0.013	94	0.008	0.036	94
5	4	4	0.004	0.000	94	0.012	0.009	87
6	4	5	0.005	0.007	92	0.014	0.009	85
7	5	3	0.027	0.038	54	0.045	0.034	50
8	5	4	0.008	0.003	87	0.013	0.008	86
9	5	5	0.007	0.036	90	0.018	0.026	82
10	6	3	0.026	0.039	56	0.055	0.025	39
11	6	4	0.005	0.011	92	0.009	0.040	92
12	6	5	0.012	0.008	81	0.031	0.031	70

**Table 7:** Shuffled frog-leaping algorithm.

Parameter	Value	Parameter	Value
Memeplex Size	7	Number of Parents	2
Number of Memeplexes	3	Number of Offspring	3
Population Size	Memeplex Size × Number of Memeplexes	Maximum Number of Iterations	5

**Table 8:** Characteristics of the genetic algorithm combined with an ANN (GA-ANN).

Parameter	Value
Max Generations	100
Recombination (%)	15
Lower/Upper Bound	[−1, 1]
Crossover (%)	50
Crossover Method	Single Point
Selection Mode	1
Population Size	150

**Table 9:** Statistics of the developed models.

Model	Train					Test					All				
	Average	STD	COV	*AAE*	*VAF*%	Average	STD	COV	*AAE*	*VAF*%	Average	STD	COV	*AAE*	*VAF*%
SFLA-ANN	1.01	0.07	0.07	0.01	0.94	1.04	0.08	0.08	0.03	0.94	1.02	0.07	0.07	0.06	0.93
GA-ANN	1.00	0.114	0.11	0.08	0.77	1.03	0.09	0.09	0.06	0.90	1.01	0.11	0.1	0.08	0.80
MLR	0.99	0.17	0.18	0.14	0. 54	1.01	0.21	0.21	0.18	0.54	0.97	0.18	0.18	0.15	0.53

## Data Availability

All the data is available within the manuscript.
